# Psychometric properties of the Gaudiebility (Enjoyment modulators) Scale for Adults of Morelia (GSAM)

**DOI:** 10.1371/journal.pone.0252543

**Published:** 2021-07-21

**Authors:** Ferrán Padrós-Blázquez, María Patricia Martínez-Medina, Karina Salud Montoya-Pérez, Rocío Montoya-Pérez

**Affiliations:** 1 Facultad de Psicología, Universidad Michoacana de San Nicolás de Hidalgo, Morelia, Michoacán, México; 2 Hospital General Dr. Miguel Silva, Secretaría de Salud, Morelia, Michoacán, México; 3 Instituto de Investigaciones Químico-Biológicas, Universidad Michoacana de San Nicolás de Hidalgo, Morelia, Michoacán, México; Aalborg University, DENMARK

## Abstract

Gaudiebility refers to the set of modulators that regulate the enjoyment experienced, there is only one scale to assess gaudiebility, and it is univariate, which is not congruent with the construct. Therefore, this work’s objective was to build a multifactorial scale and study the psychometric properties of the Gaudiebility Scale for Adults of Morelia (GSAM). 1,884 adults participated; 273 in the pilot study and 1,611 in the final study. They responded to the final version of the GSAM, with 24 items and a scale of experienced enjoyment. The adequate fit of the model of 6 related factors of the GSAM was corroborated through confirmatory factor analysis. Adequate Cronbach’s alpha values ​​were observed for the factors (*Concentration* (α = .796), *Enjoyment in Solitude* (α = .784), *Sense of Humor* (α = .760), *Imagination* (α = .740), *Interest* (α = .653) and *Irrational Beliefs* (α = .633)) and the total scale (α = .803). All the factors and the full scale showed positive relationships with the scale of experienced enjoyment. The GSAM obtained good indicators regarding its internal consistency and validity; therefore, it is concluded that the GSAM is adequate to assess gaudiebility in adults.

## Introduction

The study of how positive emotions appear and are regulated is one of the main areas of interest in Positive Psychology [1]. Wankel, in 1993 [2] points out that enjoyment can be conceived as a positive emotion. However, it should first be noted that it is essential to distinguish between what has been called basic pleasure and complex or evolved enjoyment [3]. According to Kringelbach and Berridge [4], it is possible to distinguish between the neural circuits of fundamental pleasures (basic pleasure) and superior pleasures (complex enjoyment), which overlap; that is, they share a joint neural base. Thus, complex enjoyment can be considered an evolved mechanism of basic pleasure, consistent with the idea that the more evolved systems settle on, the older ones, not replacing but overlapping [5].

From the proposal of Gómez-Hernández et al. [3], the oldest neuroanatomical areas of the brain, in a phylogenetic and ontogenetic sense, located in the most profound areas of the brain (nucleus accumbens, ventral pallidum, brainstem) would be involved in basic pleasure. Others found in the cortex (orbitofrontal, cingulate, medial prefrontal and insular) and probably with other parts of the neocortex would be the substrate of complex enjoyment experiences. A review on the neuroanatomical bases of pleasure and happiness (which would include complex enjoyment) can be seen in Kringelbach and Berridge [4, 6, 7].

According to Seligman & Csikszentmihalyi [8], both pleasure and enjoyment refer to good sensations that people experience; however, pleasure is derived from satisfying homeostatic needs (such as hunger, sex…), and in enjoyment, these sensations arise when the limits of the homeostasis are broken (e.g., doing a sports activity, tasting wine, in a stimulating conversation).

Some multidimensional models of enjoyment have been described, such as Warner’s [9] with three factors, engagement, positive affect and fulfillment. The engagement was identified as focused attention (e.g., concentration, absorption, engrossment, attention), positive affect was identified as feelings of pleasure, happiness, or contentment and fulfillment were proposed to have four aspects: meaning, reward, usefulness, and being worthwhile.

On the other hand, there is the model proposed by Davidson [10] with five (Pleasure, Relatedness, Competence/Challenge, Improvement, and Engagement), but both models allude to the enjoyment experienced after completing a list of activities. However, none of the studies distinguish between different modes of enjoyment. It should be noted that currently, there are no studies that delve into the different modes of enjoyment. The differences between the enjoyment experienced derived from the sports practice is very different from that which can be obtained by observing a work of art, and this, in turn, differs from that experienced by speaking with friends. In the present work, complex enjoyment is considered without referring to any specific model (multidimensional or unidimensional).

Gaudiebility is defined as: "*a construct that encompasses all those processes that mediate between stimuli and enjoyment (complex or evolved) that people experience*, *that is*, *the set of modulators that regulate the subjective sensations of living rewarding experiences*" [11] p 414. Three types of gaudiebility components have been distinguished: *skills* (such as imagination, sense of humor, ability to be interested), *cognitive beliefs*/*styles* (such as irrational beliefs such as the person who laughs a lot is not very competent or insensitive, or the locus of control) and lifestyle (as a trend towards active or passive leisure) [12].

Since the concept was described, a scale has been constructed with psychometric data available in Spain [11] and Mexico [13]. Gaudiebility studies have been carried out about the presence of psychopathology [14] using specific samples. Schizophrenia and substance dependence [15], antisocial disorder [16], and has been reported a moderate and positive relationship between gaudiebility and health status [17]. The relationship between gaudiebility and hypertension has also been studied [18], even with spinal cord injuries [19].

It is essential to mention that therapy has been developed to increase gaudiebility that has shown efficacy as an adjuvant treatment in depressive patients [20]. Dr. Montgomery [21] from Peru proposed Gaudiebility and Mindfulness as functional behavior therapy categories to treat experiential avoidance. Finally, the concept has received some international interest since recently; some Spanish authors have carried out a systematic review of subjective well-being and gaudiebility [22]. In this review, it was concluded that gaudibility is related to the quality of life, health, and to a lesser extent with subjective well-being [22].

As mentioned above, there is a scale to assess gaudiebility that has also been adapted for the Mexican population [13]. However, it lacks adequate construct validity because it is unifactorial, and theoretically, different factors should be distinguished. Hence, in the present investigation, the following possible factors were considered: a) skills such as "Imagination", an element that is associated with enjoyment [23, 24]; b) "Interest" which has been considered different from enjoyment [25]; c) "Sense of humor" that has been considered as a form of enjoyment since the nineteenth century [26] and, d) "Concentration" that has been indicated as a necessary element to achieve to the state of flow [27] in addition to being related to other skills such as interest.

On the other hand, "Irrational beliefs" have also been considered because, as has been suggested, beliefs about enjoyment can affect the enjoyment experienced [28]. Finally, two frequent conditions have been considered. The first is "enjoyment in solitude", note that it has been emphasized that it is a skill that is not related to social avoidance [29] and has been positively associated with mental health [30]. Using the same logic, the second frequent condition that refers to "company enjoyment" was also introduced.

Therefore, the objective of this research was to construct and study the psychometric characteristics (study of the internal structure, internal consistency, offer validity evidence and descriptive data) of the Gaudiebility Scale for Adults of Morelia (GSAM).

## Method

The present study was carried out through three studies: a) construction and content validity study, b) pilot study and, c) study of the final version.

### Construction and content validity study

#### Procedures

In the content construction and validity study, 61 items were first drawn up from the construct’s definition and considered the different potential factors to assess adults’ gaudiebility The possible factors considered were the following; "Imagination", "Interest", "Sense of humor", “Self-efficacy versus boredom”, "Irrational beliefs", "enjoy in solitude" and " Enjoy in Company, " considered as well-known candidates to be part of gaudiebility [31] in the same way as was done with the scale for children and adolescents (GSCA) [32]. Afterward, three expert judges in psychometry were asked, who were informed about the construct’s nature and its possible factors, to evaluate each item’s writing and relevance to obtain the content validity. Considering the following characteristics: representativeness, relevance, diversity, clarity, simplicity and comprehensibility [33].

#### Results

The judges approved 54 items.

### Pilot study

#### Participants

A sample of 273 adults between 18 and 79 years old (M = 34.94, SD = 13.31), of which 138 (50.4%) were men from the city of Morelia (Michoacán, Mexico), was studied. With a mean of 12.40 (SD = 4.30) years of schooling, 49.6% of the sample referred to being single, 42.35% married or in a common-law union, and 6.2% divorced or separated.

#### Instruments

Gaudiebility Scale for Adults of Morelia (GSAM). The first version of 54 items that are scored with a scale of five response options (from "Totally agree" to "Totally disagree") was applied.

#### Procedures

In the first place, the present investigation project was evaluated and approved by the ethics committee of the Faculty of Psychology of the Universidad Michoacana de San Nicolás de Hidalgo. The 54-item scale, with an approximate duration of 12 minutes, was applied to passers-by who responded voluntarily. The confidentiality of the information and the anonymity of all participants was ensured. In the administration studies of the scales, all participants signed informed consent.

#### Data analysis

The inclusion of items followed these criteria [34]: (1) factor loadings should be more significant than 0.40; (2) items of one factor should have no or minimal cross-loadings with other factors; and (3) there should be conceptual consistency among items loaded on a shared factor. Also, (4) a minimum corrected correlation of 0.30 with full scale was established as a criterion for item goodness; and (5) no increase in Cronbach’s alpha was observed if the item was eliminated [35]. Subsequently, a study of internal consistency was performed using the alpha of Cronbach of the total scale and each factor taking as reference Campo-Arias and Oviedo [36] where values equal to or greater than 0.70 are acceptable.

For the study of the internal structure, principal component analysis and oblimin rotation was used. The recommendations of Lloret-Segura et al. [37] and EFA data performed by extraction of principal axis factorization are offered, considering the Bartlett sphericity test and the Kaiser, Meyer, and Olkin (KMO) test to assess the applicability of factor analysis. For the extraction of eigenvalues, the number of factors was considered a function of values ​​higher than unity or that a vast difference was observed between the eigenvalues ​​and that they had theoretical congruence. Finally, the SPSS 22.0 Program was used to analyze the data.

#### Results

In the first place, in the Exploratory Factor Analysis, it was observed that the indices (Bartlett’s sphericity test = 5347.399; *gl* = 1431 with *p* < .001; Kaiser Meyer Olkin index = .830) indicated that the data were capable of being analyzed at through a Principal Component Analysis. After purification, the result was a scale of 24 items with a solution of 6 factors (with eigenvalues greater than unity, congruent with the theoretical framework and that explained 60.45% of the variance) see Table 1. All the items obtained had corrected correlations (excluding the item itself) with the total of the scale greater than .30, and, in no case when eliminated, did they increase the value of Cronbach’s alpha of the total scale. In addition, those items that showed a more significant factorial load in a single factor were preserved (see Table 2).

**Table 1:** Eigenvalues explained and accumulated variance of the GSAM components.

Component	Eigenvalues	% Explained variance	% Cumulative variance explained
**01**	**6.053**	**25.221**	**25.221**
**02**	**2.763**	**11.511**	**36.732**
**03**	**1.880**	**7.834**	**44.566**
**04**	**1.383**	**5.764**	**50.330**
**05**	**1.269**	**5.288**	**55.617**
**06**	**1.161**	**4.836**	**60.454**
07	.922	3.842	64.296

**Table 2:** Rotated component matrix after confirmatory factor analysis.

Ítem	Interest	Concentration	Imagination	Enjoy in Solitude	Irrational beliefs	Humor/ Laught
**7**	**.786**		.340			
**1**	**.599**					
**20**	**.455**		.342			
**12**	**.417**		.306			
**21**		**.729**				
**10**		**.671**				
**17**		**.655**				
**5**		**.620**				
**18**			**.852**			
**6**			**.790**			
**13**			**.570**			
**23**			**.517**			.322
**15**		.350		**.722**	.390	
**2**				**.658**		
**8**				**.633**	.343	
**19**				**.631**		
**16**	.323			.301	**.679**	
**4**					**.607**	
**11**					**.606**	
**24**				.308	**.542**	
**22**			.325			**.640**
**14**	.385		.304			**.628**
**9**						**.626**
**3**	.309					**.570**

*Only displays values greater than .30.

In addition, adequate reliability (Cronbach’s alpha) was observed for each of the 6 factors (composed of 4 items each): Imagination (α = .794), Irrational beliefs (α = .741), Enjoyment in solitude (α =. 738), Interest (α = .733), Concentration (α = .728) and Sense of humor, (α = .710). Finally, the value of the total scale was also acceptable (α = .858).

### Study of the final version (24 items)

#### Participants

The sample included 1,611 adults (mean, M = 27.63; SD = 11.54; min-max 18–79) of which 699 (43.4%) were female, from the city of Morelia and the state of Michoacán (Mexico). For schooling years, a mean of 12.44 was observed (SD = 4.29; min-max 0–24), 57.8% of the sample referred to being single, 35.6% married or in a common-law union, and 5.9% divorced or separated.

#### Instruments

Gaudiebility Scale for Adults of Morelia (GSAM). The data of the last study is described, where the final version of 24 questions was applied, which are scored with a scale of five response options (from "Totally agree" to "Totally disagree") with values ​​from 0 to 4. The scale’s total score is obtained by adding the items and the values ​​can range from 0 to 96. The GSAM has 6 subscales (made up of 4 items each): 1) "Imagination" (6,13,18 and 23), 2) "Irrational beliefs" (4,11,16 and 24), 3) "Enjoyment in solitude" (2,8,15 and 19), 4) "Interest" (1,7,12 and 20), 5) "Concentration" (5,10,17 and 21) and, 6) "Sense of humor" (3,9,14 and 22). Items 2,4,5,8,10,11,15,16,17,19, 21 and 24 are inverse.

Experienced Enjoyment Scale for Adults (EESA) made by Padrós-Blázquez & Martínez-Medina [38]: consisting of 6 items with five response options: ("Totally disagree" (0), "Somewhat Disagree" (1), "Neither agree nor disagree" (2), "Somewhat agree "(3) and “Totally agree "(4) has a single factor and the Cronbach’s alpha value of the scale is adequate .856, in the present study it was .851. High scores indicate a high level of enjoyment experienced [38].

#### Procedures

The scale with 24 items was administered to adults on public roads and agreed to respond voluntarily and anonymously to the scale. The participants also had to sign an informed consent and provide data on their age and sex. 268 adults were asked to respond to GSAM and EESA. The application of the instruments required approximately 7 minutes.

#### Data analysis

CFA was carried out through analysis of structural equations and the robust maximum likelihood method (Robust ML) was used to assess the 6-factor model with a second-order model and the 6-factor related model. The following indices were used to evaluate the fit of the models. The chi-square statistic (χ2) of Satorra-Bentler [39], whose non-significance indicates a good fit, values χ2 / gl> 5. Also the Comparative Fit Index (CFI), the Non-normed Fit Index (NNFI) and the Index of Normed fit (NFI) whose values > .90 indicate adequate fit and > .95 excellent. Finally, the index, Square Root of the Mean Quadratic Error (RMSEA) and its corresponding Confidence Interval of 90% (90% CI) were used; values < .08 indicate a good fit and < .05 excellent [40, 41].

For the descriptive, the mean, standard deviation, skewness, kurtosis and percentiles were used. Cronbach’s alpha was used to study reliability. Likewise, the corrected correlation (between the item and the total scale, without adding the item itself) and the value of Cronbach’s Alpha of the total scale were used if the item is eliminated. The concurrent validity evidence of the GSAM was explored through Pearson’s correlation with the experienced enjoyment scale (EESA). It was expected to find positive correlations between gaudiebility and the enjoyment experienced and somewhat lower correlations with each of its factors and the EESA.

Analyzes were carried out with the SPSS 22.0 program [42], except for the AFC, which used the EQS 6.1 program [43, 44].

#### Results

The items’ mean ranged between 2.05 (item 10) and 3.38 (item 12), while the standard deviation values were distributed between 0.939 and 1.386. The asymmetry and kurtosis values indicate that most of the items are distributed following the normal law. The correlation of some items with the corrected total score (without taking into account the item itself) is below .30 (and above .20), but no item, when eliminated, increases the Cronbach’s alpha value of the full scale (Table 3). It is essential to mention that all items correlated above .35 with the corrected score of their factor, and all contribute significantly to the alpha value of their factor.

**Table 3:** GSAM item analysis. Mean, standard deviation, skewness, kurtosis of each item are presented. Likewise, each item’s Pearson correlation with the corrected full scale (excluding the item itself) and Cronbach’s alpha without considering the item.

Ítem	Mean	SD	Skewness	Kurtosis	Correlation with full scale (corrected)	Full-scale alpha if the item is removed
1	3.11	0.95	-1.15	1.14	.34	.80
2	2.34	1.37	-0.29	-1.15	.31	.80
3	3.11	1.04	-1.16	0.75	.31	.80
4	2.95	1.22	-0.86	-0.39	.34	.80
5	2.42	1.39	-0.33	-1.21	.37	.79
6	2.89	1.15	-0.87	-0.07	.30	.80
7	3.03	0.96	-0.95	0.61	.45	.79
8	2.77	1.23	-0.71	-0.50	.44	.79
9	2.40	1.37	-0.38	-1.04	.24	.80
10	2.05	1.31	0.02	-1.14	.27	.80
11	2.81	1.29	-0.71	-0.73	.28	.80
12	3.38	0.94	-1.77	1.60	.38	.79
13	2.77	1.11	-0.67	-0.22	.28	.80
14	3.17	1.02	-1.21	0.91	.39	.79
15	2.76	1.22	-0.67	-0.57	.45	.79
16	3.23	1.14	-1.34	0.78	.44	.79
17	2.39	1.32	-0.28	-1.08	.29	.80
18	2.78	1.18	-0.72	-0.39	.32	.80
19	2.72	1.29	-0.63	-0.77	.38	.79
20	3.28	0.97	-1.47	1.80	.36	.79
21	2.49	1.29	-0.37	-1.01	.41	.79
22	2.75	1.15	-0.67	-0.39	.26	.80
23	2.56	1.29	-0.54	-0.79	.26	.80
24	2.86	1.24	-0.79	-0.45	.41	.79

**Fig 1:**
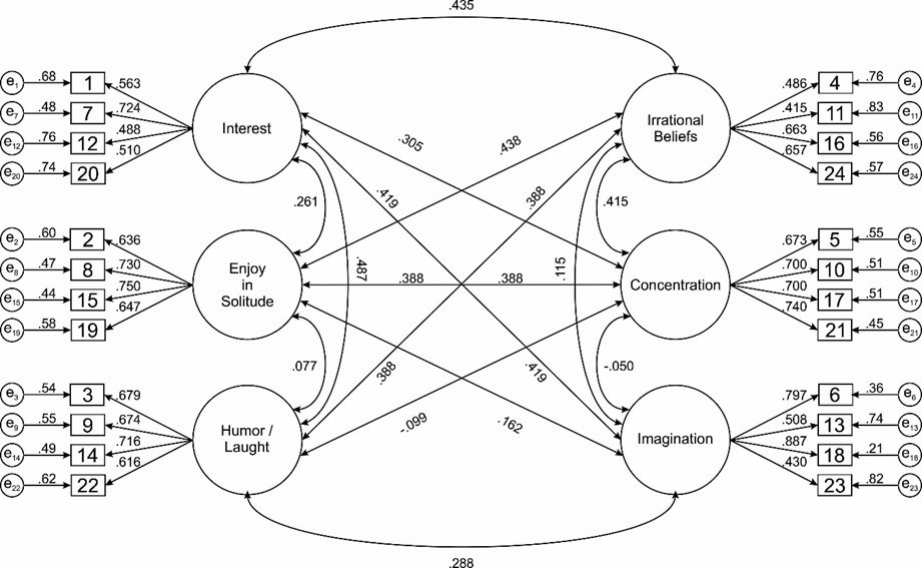
GSAM 6-factor related model.

*Internal structure*. Through the AFC, it was confirmed that the 6-factor structure was adequate. Specifically, it was observed that the model with 6 related factors presents an acceptable fit and better than the model with six factors and one of the second order. Regarding the Satorra-Bentler χ2, both were significant; however, the value of χ2 / gl; in both cases, the values were < .05. On the other hand, the other NFI, NNFI and CFI indices were adequate for the 6-factor model but not for the 6-factor model and a second-order model. Finally, the RMSEA was adequate for both models (Table 4). It can be concluded that the results obtained suggest adequate goodness of fit for the model of 6 related factors (F1: Interest, F2: Enjoyment in solitude, F3: Sense of humor, F4: Irrational beliefs, F5: Concentration and F6: Imagination (Fig 1).

**Table 4:** The models’ fit indices are based on the confirmatory factor analysis of the 6-factor model related to a second-order factor and the 6-factor model related.

Modelo	χ^2^(gl)	χ2/gl	NFI	NNFI	CFI	RMSEA (90% IC)
1 second.order factor	1140.81 (246)	4.64	.873	.885	.897	.048 (.045:.050)
**6 related**	**787.75 (237)**	**3.32**	**.912**	**.926**	**.937**	**.038 (.035:.041)**

Indices; χ2 = chi-square; gl = degrees of freedom; χ2 / gl; chi-square divided by the degrees of freedom; NNFI = Non-normed fit index; NFI = Normed fit index; CFI = Comparative Adjustment Index; RMSEA = Square Mean Approximation Error; CI = Confidence Interval.

*Evidence of validity*. Evidence on concurrent validity was assessed using Pearson’s correlations between the GSAM (and its factors) and the EESA. All the correlations between the EESA and the different factors and the GSAM total score were significant and positive. Interest, humor, irrational beliefs and the total score of GSAM showed moderate correlations. Correlations of the factors: concentration, imagination and enjoyment in solitude were low (Table 5).

**Table 5:** Pearson’s correlations between the full scale (GSAM) and its factors with the EESA.

Scale	Interest	Loneliness	Humor	Beliefs	Concentration	Imagination	GSAM
**EESA**	.560**	.225**	.465**	.416**	.276**	.238**	.550**

*p < .01

**p < .001

*Internal consistency*. The Cronbach’s alpha value of the total scale was adequate (α = .803), also that of the following four factors: *Concentration* (α = .796), *Enjoyment in Solitude* (α = .784), *Sense of Humor* (α = .760) and *Imagination* (α = .740). *Interest* (α = .653) and *Irrational Beliefs* (α = .633) were acceptable.

*Normative data*. The mean was 67.02, the SD = 12.14, the mode was 69, the minimum value was 2 and the maximum was 96. The asymmetry and kurtosis values were -0.413 and 0.692, which indicates that the total score of the GSAM follows a normal distribution. Table 6 shows the percentiles.

**Table 6:** Description of the GSAM percentiles.

Percentile	Total
1	39.0
10	51.0
20	57.0
25	59.0
30	61.0
40	65.0
50	68.0
60	70.0
70	74.0
75	75.0
80	78.0
90	83.0
99	91.0

## Discussion

This work’s objective was to construct and analyze some of the psychometric properties (internal structure, internal consistency, the goodness of the items and evidence of validity) of a multifactorial instrument to assess adults’ gaudiebility. The AFC corroborated the internal structure of 6 related factors (Concentration, Enjoyment in Solitude, Sense of Humor, Imagination, Interest and Irrational Beliefs). It is crucial to bear in mind that gaudiebility is conceived as a set of modulators that regulate the enjoyment experienced [8]; therefore, it was to be expected that the scale would be multifactorial and not unifactorial like the previous scale [11, 13].

Although some items obtained low corrected correlations with the total scale, all contribute positively to the value of the total alpha of the scale and, above all, were adequate for its factor. The reliability indices of the GSAM and each of the factors were satisfactory.

The correlations found between the GSAM and each factor with the scale of experienced enjoyment were positive and moderate, especially with the full scale, consistent and expected. It should be remembered that gaudiebility is the set of modulators of enjoyment, not enjoyment itself; theoretically, the higher the gaudiebility, the more likely it is to experience enjoyment. Therefore, moderate correlations were expected, but not excessively high, since they are related concepts, and they are not synonymous.

However, the low correlations observed between the enjoyment experienced and the factors imagination, concentration and enjoyment in the solitude of GSAM could be explained because the scale of experienced enjoyment is generic. People, when responding regarding the enjoyment experienced, may have in mind those more prototypical modalities such as those carried out in company and activities that involve high arousal (such as playing sports, laughing, going up to a theme park attraction, etc.) and may not have less prototypical modes of enjoyment in mind (such as the enjoyment experienced in reading a book, looking at a painting, having a conversation, etc.). Therefore, it would be desirable in future research to study the relationship of the GSAM with an instrument that distinguishes between modes of enjoyment.

Regarding the normative data, the average score is well above the theoretical midpoint, as was the case with the previous scale in the Spanish [11] and Mexican [13].

### Limitations and suggestions for future studies

It is essential to point out some limitations of the present work; first, the validity evidence is scarce. It is necessary to study the relationship of the GSAM with other variables, for example, positive and negative affect, satisfaction with life, psychological well-being, depressive symptoms, among others. Nor has temporal stability been studied because conceptually, gaudiebility includes modulators such as skills and beliefs; it would be expected that the scores would be maintained over time, but this should be corroborated in future research. Likewise, it would be convenient to study the sensitivity to change, for example, after successfully applying an intervention to increase gaudiebility, such as that applied to patients with depression [20].

On the other hand, an important variable that was not controlled and can influence the results is social desirability. Future research recommends studying the incidence of this variable on GSAM scores and its factors.

Regarding the generalization of the results, it should be noted that the samples have only been obtained from the adult population of Michoacán, which is a state of Mexico; it would be convenient to replicate the study in other regions. Likewise, it is desirable to adapt and study the psychometric properties of the GSAM in other countries and observe if the results are corroborated.

It is also important to mention that if demographic variables such as sex and age are considered, it would be of great interest to carry out invariance analysis in order to evaluate the behavior of each of the scale items based on these variables.

It can be concluded that the Gaudiebility Scale for Adults of Morelia obtained good indicators regarding its validity and reliability. The GSAM can be helpful as an instrument to assess the modulators of enjoyment, which can be considered an indicator of people’s optimal functioning.

## Supporting information

S1 File (DOCX)

S2 File (XLSX)
