# Supplementary material for: Psychometric properties of the Gaudiebility (Enjoyment modulators) Scale for Adults of Morelia (GSAM)
Source: PLoS One. 2021 Jul 21;16(7):e0252543. doi: 10.1371/journal.pone.0252543 (PMC8294498; doi:10.1371/journal.pone.0252543)
Supplement: S1 File — (DOCX) [file pone.0252543.s001.docx]

**EGM-24**

**Gaudiebility Scale for Adults of Morelia**

**Instructions:** This scale is designed to know your opinion and the situations in which you may experience a sense of enjoyment. Please answer each question by marking a cross (x). Your answer should indicate how much you agree with each statement, not what you think it should be or what you would like. Therefore, answer quickly and do not analyze your answers too much, since the first one is usually the most correct. Try to answer all the questions and not miss any.

| **Indicate the level of agreement in each of the following statements:** | **Strongly disagree** | **Somewhat disagree** | **Neither agree or disagree** | **Somewhat agree** | **Strongly agree** |
| --- | --- | --- | --- | --- | --- |
| 1. Most of the activities I do seem interesting to me. | 0 | 1 | 2 | 3 | 4 |
| 2. When I'm alone I get bored easily. | 0 | 1 | 2 | 3 | 4 |
| 3. Many things make me laugh. | 0 | 1 | 2 | 3 | 4 |
| 4. If you laugh a lot, your performance drops. | 0 | 1 | 2 | 3 | 4 |
| 5. I have a hard time concentrating, even when they tell me stories that interest me. | 0 | 1 | 2 | 3 | 4 |
| 6. I am a very imaginative person. | 0 | 1 | 2 | 3 | 4 |
| 7. I am easily interested in most things I do. | 0 | 1 | 2 | 3 | 4 |
| 8. I hardly ever have fun when I'm alone. | 0 | 1 | 2 | 3 | 4 |
| 9. My friends tell me that I laugh a lot. | 0 | 1 | 2 | 3 | 4 |
| 10. I lose concentration easily when doing work. | 0 | 1 | 2 | 3 | 4 |
| 11. I believe that to have fun you need to have money. | 0 | 1 | 2 | 3 | 4 |
| 12. I usually enjoy things that I find interesting to the fullest. | 0 | 1 | 2 | 3 | 4 |
| 13. I have a good time when I imagine. | 0 | 1 | 2 | 3 | 4 |
| 14. I am used to laughing several times a day. | 0 | 1 | 2 | 3 | 4 |
| 15. I have a hard time having fun when I'm alone. | 0 | 1 | 2 | 3 | 4 |
| 16. I think people who laugh a lot are stupid. | 0 | 1 | 2 | 3 | 4 |
| 17. People tell me that I lose concentration easily. | 0 | 1 | 2 | 3 | 4 |
| 18. I have a lot of imagination. | 0 | 1 | 2 | 3 | 4 |
| 19. At home, when my family is away, I get bored easily. | 0 | 1 | 2 | 3 | 4 |
| 20. If I put my mind to it, I am able to find interest in whatever activity I do. | 0 | 1 | 2 | 3 | 4 |
| 21. I have a hard time concentrating, even with something that interests me. | 0 | 1 | 2 | 3 | 4 |
| 22. I laugh easily when others make a joke. | 0 | 1 | 2 | 3 | 4 |
| 23. If I put my mind to it, I can easily make up stories. | 0 | 1 | 2 | 3 | 4 |
| 24. I think that people who laugh a lot are not very aware of the problems of the world. | 0 | 1 | 2 | 3 | 4 |
